# Transpedal Retrograde Recanalisation of a Dorsalis Pedis Artery Occlusion Associated With Arteriovenous Fistula After Combined Arthroscopic and Mini Open Talocalcaneotibial Arthrodesis

**DOI:** 10.1016/j.ejvsvf.2026.03.002

**Published:** 2026-03-16

**Authors:** Michael Czihal, Sebastian Baumbach, Hans Polzer, Sinan Deniz

**Affiliations:** a Division of Vascular Medicine, Medical Clinic and Policlinic IV, LMU Hospital, Munich, Germany; b Department of Orthopaedics and Trauma Surgery, Musculoskeletal University Centre Munich (MUM), Munich, Germany; c Department of Radiology, Ludwig Maximilians University, LMU University Hospital, Munich, Germany

**Keywords:** Angioplasty, Arthrodesis, Fistula, Retrograde tibial access

## Abstract

**Objective:**

Arterial injury following hindfoot fusion is an exceptionally rare but serious complication that may result in limb threatening ischaemia.

**Methods:**

This report presents the case of a 71 year old man who experienced acute foot ischaemia after undergoing combined arthroscopic and mini-open tibiotalocalcaneal arthrodesis. Angiography revealed anterior tibial artery occlusion with an arteriovenous fistula. After failed antegrade attempts, transpedal retrograde recanalisation via the dorsalis pedis artery successfully restored perfusion and excluded the fistula.

**Results:**

This case highlights the potential for arterial injury as a rare complication of complex hindfoot procedures. It underscores the importance of multidisciplinary collaboration between orthopaedic and endovascular specialists and demonstrates that minimally invasive recanalisation techniques can effectively salvage the limb and prevent major amputation.

## Highlights

• A rare case of arterial injury after complex hindfoot arthrodesis
• Post-operative foot ischaemia caused by dorsalis pedis artery occlusion and AV fistula
• Retrograde transpedal recanalisation with successful restoration of foot perfusion
• Endovascular management prevented major amputation
• Interdisciplinary pre- and peri-operative workflow is essential

## INTRODUCTION

Complex foot and ankle surgery, including hindfoot correction procedures such as open or arthroscopic talocalcaneotibial arthrodesis, carries a risk of post-operative complications.^1^ However, arterial injury has rarely been described as a complication. This case report highlights the role of modern endovascular techniques in restoring blood flow in the setting of post-operative foot ischaemia. Furthermore, the potential value of an interdisciplinary setting with detailed pre-operative perfusion assessment in patients at risk of arterial complications is discussed.

## CASE REPORT

A 71 year old man, a former miner, who had experienced an open ankle fracture 40 years earlier, presented for surgical treatment of disabling post-traumatic osteoarthritis of the right hindfoot. Comorbidities included atrial fibrillation, moderate chronic obstructive pulmonary disease (GOLD stage II, Global Initiative for Chronic Obstructive Lung Disease), well controlled type 2 diabetes mellitus (glycated haemoglobin level, 5.7%), and arterial hypertension. The patient had no known history of peripheral arterial occlusive disease and denied symptoms of claudication. No formal pre-operative vascular assessment, such as ankle brachial index or duplex ultrasound, had been performed; however, a palpable right dorsalis pedis pulse was present.

**Figure 1:**
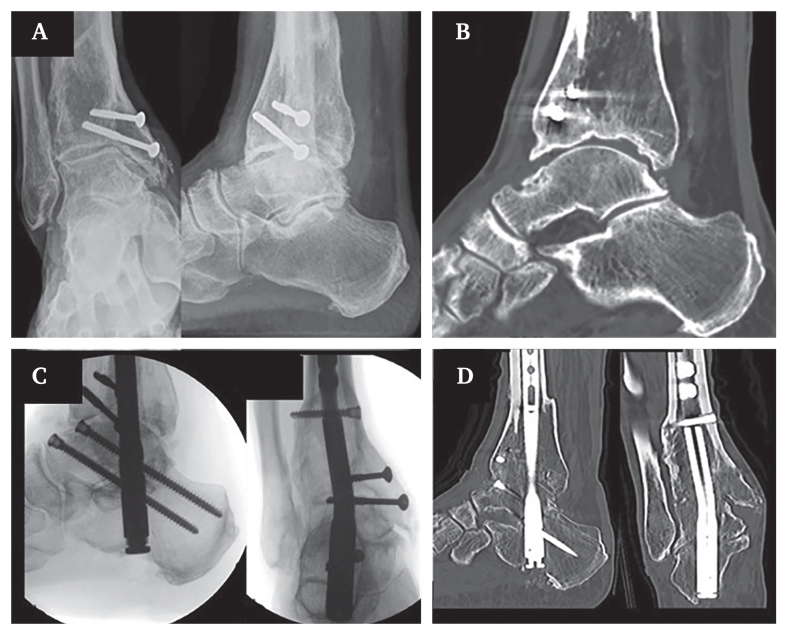
(A) Pre-operative radiographic and (B) computed tomographic imaging of the right hindfoot documenting end stage, multiplanar varus osteoarthritis of the ankle and the subtalar joint. (C) Intra-operative radiographic and (D) post-operative computer tomography imaging revealing good bony contact at the arthrodesis sides and an acceptable hindfoot alignment.

Pre-operative imaging (Fig. 1A and B) revealed end stage, multiplanar varus osteoarthritis of the ankle and the subtalar joint. Given his high physical activity level and history of an open fracture, the patient opted for arthrodesis. Because of the above outlined comorbidities and the complex deformity, a combined arthroscopic (ankle joint) and mini open (sinus tarsi) tibiotalocalcaneal arthrodesis was performed using a retrograde ankle fusion nail (Wright Medical, Memphis, TN, USA). The procedure was carried out with the patient in supine position, and distal locking screws were inserted from anterior to posterior. Anterior locking bears the risk of injuring the anterior neurovascular bundle. Because the procedure was performed arthroscopically, only cutaneous step incisions were made, requiring blunt prepping down onto the talus and using the drilling guides for drilling and screw insertion.^2^ Intra- and post-operative imaging (Fig. 1C and D) demonstrated good bony contact at the fusion sites and an acceptable hindfoot alignment.

**Figure 2:**
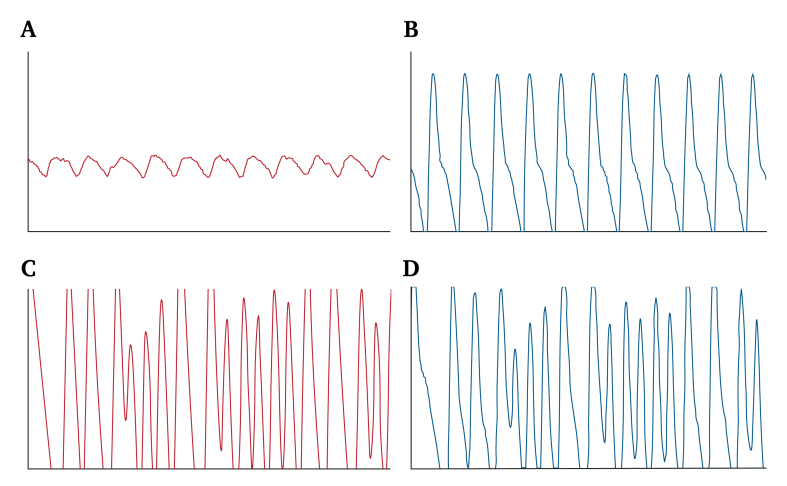
Pulse volume recordings of the left (red) and right (blue) forefoot (A), (B) before and (C), (D) after retrograde recanalisation of the right dorsalis pedis artery. Significant perfusion improvement of the right forefoot after the procedure.

**Figure 3:**
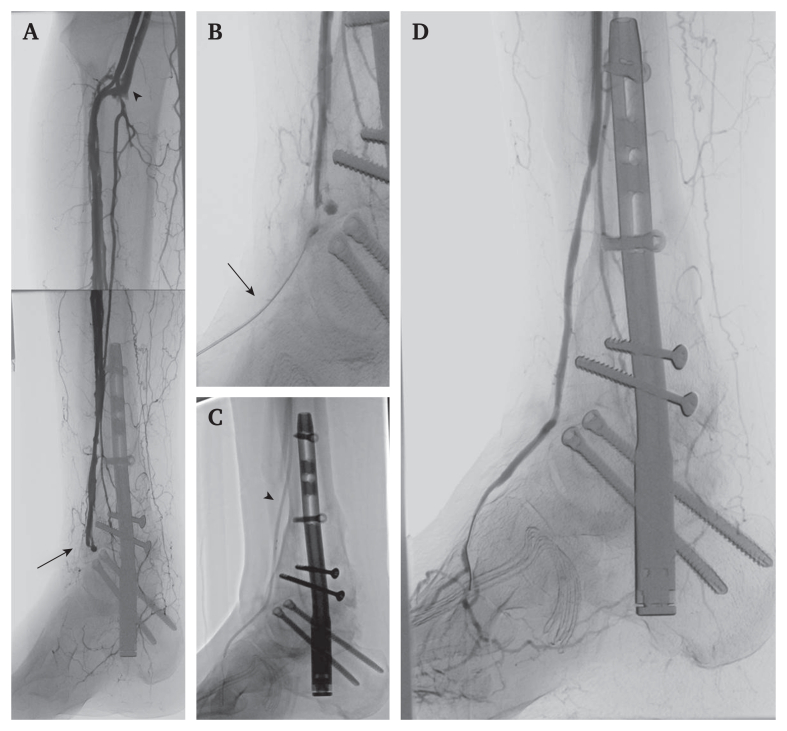
(A) Digital subtraction angiography revealing occlusion of the dorsalis pedis artery (arrow) and arteriovenous fistula (arrowhead). (B) Retrograde puncture of the dorsalis pedis artery (arrow). (C) Balloon angioplasty of the anterior tibial and dorsalis pedis artery (arrowhead: 2.5/2.0 mm inflated tapered balloon catheter). (D) Final angiographic result documenting restored patency of the dorsalis artery and closure of the arteriovenous fistula.

Acute ischaemic symptoms were noted immediately after surgery. Segmental pulse volume recordings demonstrated severely impaired perfusion (Fig. 2). In view of the acute limb threatening ischaemia with absent pulses, the patient was transferred directly to the angiography suite. Digital subtraction angiography was chosen over computed tomography angiography to allow immediate diagnosis and, if feasible, endovascular treatment without delay. Digital subtraction angiography was performed within two hours, via an ipsilateral antegrade femoral approach, revealing chronic total occlusion of the posterior tibial artery. The acute ischaemia was caused by occlusion of the large calibre anterior tibial artery at the upper ankle joint (Fig. 3A, arrow) and a haemodynamically significant arteriovenous fistula to the anterior tibial veins (Fig. 3A, arrowhead). Collateral flow from the peroneal artery was insufficient owing to shunting through the arteriovenous fistula. Possible mechanisms included thrombotic occlusion from vessel stretching and localised arterial compression by distal locking screws.

After several failed attempts at antegrade guidewire passage, the dorsalis pedis artery was punctured distal to the occlusion using a micropuncture introducer access set and a 2.9 F sheath was inserted (Cook Medical, Bloomington, IN, USA) (Fig. 3B). Retrograde guidewire passage was successful with a 0.018 inch guidewire (V18 Control, Boston Scientific, Marlborough, MA, USA), which was then externalised through the inguinal sheath. Angioplasty of the dorsalis pedis artery was performed with a 3.0 × 40 mm standard balloon catheter. Although the balloon was inflated, the dorsalis pedis artery was rewired via the balloon catheter in an antegrade direction with a 0.014 inch wire. Further balloon angioplasty was performed using a 2.5/2.0 × 210 mm tapered balloon catheter, also covering the pedal puncture site (Fig. 3C). Final angiography documented restored, brisk flow to the foot, as well as exclusion of the arteriovenous fistula (Fig. 3D). The occlusion of the posterior tibial artery was considered chronic. As brisk antegrade flow to the foot was restored via the anterior tibial artery and perfusion parameters normalised, no additional intervention on the posterior tibial artery was performed. Inguinal and pedal haemostasis were achieved without complication. Pain relief correlated with normalisation of pulse volume recordings. Post-procedural anticoagulation was continued according to the patient's pre-existing indication for atrial fibrillation. No additional long term antiplatelet therapy was initiated. Wound healing was complete within a few weeks, and at one year follow up, patency of the anterior tibial artery was maintained.

## DISCUSSION

The true incidence of foot perfusion impairment after complex fractures and surgical procedures of the ankle has not been determined so far. However, in this context, arterial injuries carry the risk of potentially devastating consequences, including major amputations.^3^^,^^4^

Hindfoot fusion is a salvage procedure often performed in patients with multiple comorbidities, as in the present case. With increasing age and in the presence of cardiovascular risk factors, the prevalence of occlusive peripheral arterial disease (PAD), particularly affecting the crural and pedal arteries, can be expected to be substantial. Based on this experience, an interdisciplinary pre-operative workup was established for patients undergoing complex hindfoot surgery. This includes vascular physical examination, ankle brachial index, segmental pulse volume recordings, and colour duplex sonography of the ankle arteries. When non-invasive assessment reveals evidence of occlusive PAD with impaired foot perfusion, invasive angiographic evaluation is acquired. In PAD cases with normal foot perfusion (e.g., one patent and one occluded tibial artery), patients are also advised to undergo digital subtraction angiography. It must be stressed that the potential benefit of routine invasive assessment before complex ankle surgery in patients at risk remains to be validated, and the risk of procedure related complications such as bleeding and arterial dissection must be taken into consideration. Moreover, it is likely that, in some cases, the blood supply to the foot cannot be optimised owing to poor pedal outflow or microangiopathy. Nonetheless, pre-operative vascular information can guide surgical planning to avoid arterial injury. For instance, in this case, distal locking screws could have been inserted posteriorly, thereby reducing the risk of injuring the dorsalis pedis artery. Although direct arterial transection was unlikely, acute thrombosis and functional occlusion of the anterior tibial artery may have resulted from mechanical stretching or localised compression during nail insertion and distal locking screw placement. The arteriovenous fistula probably developed owing to minor vascular trauma in proximity to the screws.

An interdisciplinary approach is also important in the peri-operative setting. Rapid transfer to the Vascular Unit, alongside modern endovascular techniques, most probably saved the patient from major amputation. The access strategy was guided by the acute limb threatening ischaemia and the angiographic findings. After unsuccessful antegrade attempts to cross the occlusion, a transpedal retrograde approach via the dorsalis pedis artery was chosen to facilitate lesion crossing and enable prompt revascularisation within the same session. This strategy allowed simultaneous treatment of the arterial occlusion and functional exclusion of the arteriovenous fistula without the need for additional access sites. Retrograde recanalisation techniques are increasingly being incorporated in the treatment of complex femoropopliteal and below the knee chronic total occlusions in severe PAD.^5^ These endovascular techniques should also be considered in acute post-traumatic foot ischaemia when antegrade recanalisation fails, as shown here.

When performing retrograde hindfoot fusion, care should be taken to avoid vascular injury during distal locking screw placement. In this case, the retrograde transpedal recanalisation approach allowed safe crossing of the anterior tibial artery occlusion beyond the proximal trifurcation and simultaneous exclusion of the arteriovenous fistula, demonstrating a technically demanding but effective strategy in acute post-traumatic ischaemia.

This experience supports selective pre-operative vascular assessment in high risk patients undergoing complex hindfoot arthrodesis, particularly elderly patients with diabetes with previous trauma or deformity. Non-invasive assessments such as ankle brachial index, duplex sonography, and pulse volume recordings can help identify patients who may benefit from invasive angiographic evaluation before surgery.

## CONCLUSION

Modern endovascular methods, including retrograde recanalisation, are feasible in the setting of arterial injury following complex foot and ankle surgery. An interdisciplinary approach facilitates standardised pre- and post-operative workflows that can improve patient outcomes.

## ETHICAL STATEMENT

Written informed consent for publication of this case report and accompanying images was obtained from the patient. According to institutional policy and national regulations, ethical approval and institutional review board review were not required for a single case report.

## PROCESS GUIDELINE STATEMENT

This case report is presented in accordance with the PROCESS (Preferred Reporting Of CasE Series in Surgery) guideline.

## CONFLICT OF INTEREST

The authors have no competing interests.
